# 1705. Filamentous Hemagglutinin Polyclonal Antibodies Protect against Multidrug resistant Gram-negative bacteria

**DOI:** 10.1093/ofid/ofac492.1335

**Published:** 2022-12-15

**Authors:** Eman Youssef, Sondus Alkhazraji, Shakti Singh, Teclegiorgis Gebremariam, Ashraf Ibrahim

**Affiliations:** The LUndquist Institute, Torrance, California; The Lundquist Institute at Harbor-UCLA Medical Center, Torrance, California; The Lundquist Institute at Harbor-UCLA Medical Center, Torrance, California; The Lundquist Institute at Harbor-UCLA Medical Center, Torrance, California; The LUndquist Institute, Torrance, California

## Abstract

**Background:**

Multidrug-resistant (MDR) Gram-negative bacteria (GNB) are among the major healthcare-associated infections and have high mortality especially in immunocompromised patients. *Acinetobacter baumannii* (AB) and *Pseudomonas* aeruginosa (PA) are classified as “critical organism” by CDC. Therefore, new anti-infective therapies are critically needed. Our studies showed that passive immunization using antibodies against *Candida* Hyr1 peptide#5 are protective in AB pneumonia mouse model and this was attributed to the structure homology between *Candida* Hyr1 and AB Filamentous hemagglutinin protein B (FhaB). In this study, we identified FhaB epitopes that shared sequence homology with *Candida* Hyr1 peptide#5. We aim to evaluate antibody-based therapy targeting these epitopes against AB and PA infection.

Designing and evaluating FhaB peptides that share sequence similarity to Hyr1#5

**Figure:**
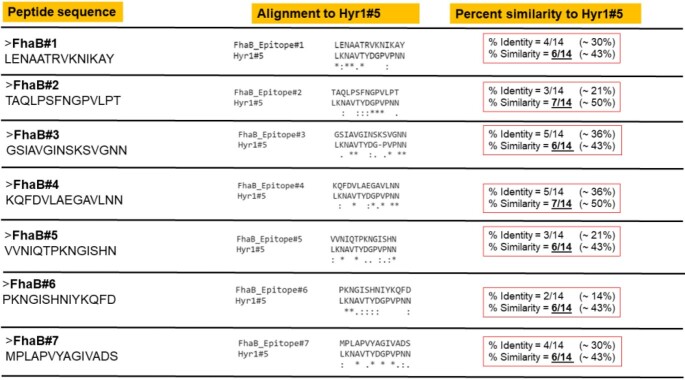

In vivo protection of FhaB antibodies in mouse model on different MDR GNB A) AB B)PA

**Figure:**
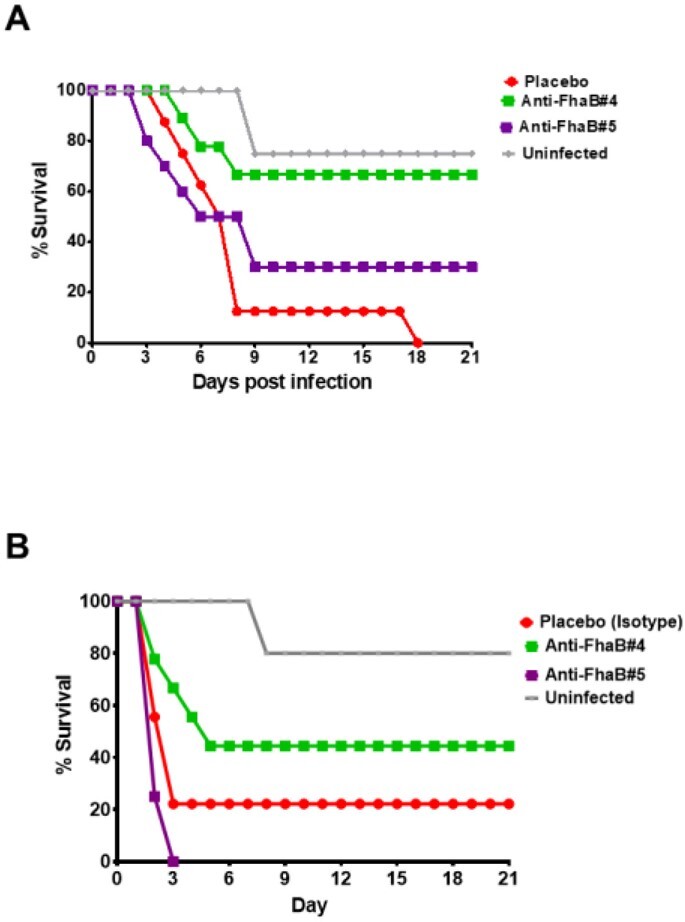

**Methods:**

Sequence homology was done using BLAST online tool. FhaB peptides and their polyclonal antibodies (pAbs) were commercially produced. Indirect ELISA was done to test recognition FhaB peptides to Hyr1#5 Abs. Binding ability of FhaB pAbs was tested against AB and PA using flow cytometry. The efficacy of pAbs in protecting against AB- or PA-induced pneumonia was studied in immunosuppressed CD1 mice by administering 30 µg of pAbs (i.p.) on Day +1 relative to infection. Survival of mice served as an endpoint.

**Results:**

We identified seven FhaB peptides that shared ∼50% sequence homology with Hyr1#5. These peptides are conserved among many GNB including AB and PA. Two peptides (FhaB#4 & FhaB#5) showed strong binding to Hyr1 Abs in ELISA. The pAbs generated against these two peptides showed ∼90% and 50% binding to AB and PA, respectively. Finally, pAbs targeting FhaB#4 protected mice from lethal dose of both AB and PA with 70% and 40% survival efficacies, respectively (p< 0.05).

**Conclusion:**

We used FhaB to generate protective pAb against MDR AB and PA. Our results warrant the further development of these Ab as novel immunotherapeutics against MDR GNB.

**Disclosures:**

**All Authors**: No reported disclosures.

